# Association between distress and knowledge among parents of autistic children

**DOI:** 10.1371/journal.pone.0223119

**Published:** 2019-09-26

**Authors:** Afiqah Yusuf, Iskra Peltekova, Tal Savion-Lemieux, Jennifer Frei, Ruth Bruno, Ridha Joober, Jennifer Howe, Stephen W. Scherer, Mayada Elsabbagh

**Affiliations:** 1 Department of Psychiatry, McGill University, Montreal, Quebec, Canada; 2 Department of Neurology and Neurosurgery, McGill University, Montreal, Quebec, Canada; 3 Autism Spectrum Disorders Research Program, Research-Institute of the McGill University Health Centre, Montreal, Quebec, Canada; 4 Research Program on Psychotic and Neurodevelopmental Disorders, Douglas Mental Health University Institute, Montreal, Quebec, Canada; 5 The Centre for Applied Genomics, Hospital for Sick Children, Toronto, Ontario, Canada; 6 McLaughlin Centre and Department of Molecular Genetics, University of Toronto, Toronto, Ontario, Canada; 7 Azrieli Centre for Autism Research, Montreal Neurological Institute, Montreal, Quebec, Canada; Chinese Academy of Medical Sciences and Peking Union Medical College, CHINA

## Abstract

Understanding the overall utility of biological testing for autism spectrum disorder (ASD) is essential for the development and integration of biomarkers into routine care. One measure related to the overall utility of biological testing is the knowledge that a person has about the condition he/she suffers from. However, a major gap towards understanding the role of knowledge in overall utility is the absence of studies that have assessed knowledge of autism along with its predictors within a representative sample of families within the context of routine care. The objective of this study was to measure knowledge of ASD among families within the routine care pathway for biological testing in ASD by examining the association between knowledge with potential correlates of knowledge namely sociodemographic factors, parental stress and distress, and time since diagnosis among parents whose child with ASD is undergoing clinical genetic testing. Parents of a child diagnosed with ASD (*n* = 85, *M*_age_ = 39.0, *SD* = 7.7) participating in an ongoing prospective genomics study completed the ASD Quiz prior to undergoing genetic testing for clinical and research purposes. Parents also completed self-reported measures of stress and distress. Parent stress and distress was each independently correlated with knowledge of ASD, *r*s ≥ 0.26, *p*s < 0.05. Stepwise regression analysis revealed a significant model accounting for 7.8% of the variance in knowledge, *F* (1, 82) = 8.02, *p* = 0.006. The only factor significantly associated with knowledge was parental distress, β = 0.30, *p* = 0.006. Parental stress, time since diagnosis, and sociodemographic factors were not significant predictors in this model. We concluded that families require tailored support prior to undergoing genetic testing to address either knowledge gaps or high distress. Ongoing appraisal of the testing process among families of diverse backgrounds is essential in offering optimal care for families undergoing genetic testing.

## Introduction

Biomarkers are indicators of a biological state that are measurable, associated with a condition, and stable across individuals [1]. It is hoped that biomarkers for autism spectrum disorder (ASD) could improve upon the identification of and intervention for ASD [2–4]. Biomarker discovery is a major priority in ASD research, with advances being made in understanding the underlying neurobiological mechanisms of ASD. For example, brain function [2] and proteomic profiles like serum homocysteine [5] have shown promising results as candidate biomarkers. It is hoped that biomarker discovery can be integrated into biological testing that could yield benefits for affected individuals and their families[6].

Currently, biological testing is integrated as part of the routine diagnostic care pathways for ASD. A care pathway describes the essential steps of health or social care centered on a person, with a specific condition, and extending across specialties and/or settings [7, 8]. For ASD, the typical care pathway leading to a diagnosis, i.e. the routine diagnostic care pathway. consists of genetic testing to rule out the presence of specific single-gene disorders, like Fragile X Syndrome and Tuberous Sclerosis [9, 10]. In some cases, it may also include metabolic testing or neurological tests. As a result of developments in our understanding of the genetic architecture of ASD [11], chromosomal microarray (CMA) testing has also been integrated into clinical practice to provide an etiological explanation for ASD [12].

The genetic architecture of ASD is complex, with numerous underlying genetic etiologies [13]. The advent of CMA testing has allowed us to detect copy number variants (CNVs) i.e. segments of the DNA that vary in copy number. CNVs are thought to explain between 7% to 18% of ASD cases [11, 14, 15]. However, different CNVs have different penetrance; some CNVs, like the 15q11–q13 duplication, are associated with severe phenotypes such as seizures, “hypotonia, global developmental delays with specific deficits in speech and language” and severe intellectual disability [16]. Other mutations have less penetrance and most likely have an impact in combination with other factors [17].

Despite this incomplete penetrance, CMA still provides some degree of clinical utility and is currently used in clinical services. CMA provides three categories of results: 1) *“abnormal”*, when an identified CNV is associated with known genetic syndromes, is *de novo*, and/or is large, (e.g. the 15q11-q13 duplication is both associated with ASD epilepsy [16], which prompts for both the monitoring of seizures and provides a likely genetic cause for the ASD), 2) *“normal”*, when either no clinically significant CNV was found or any identified CNVs are known to benign, and 3) “*variant of uncertain clinical significance (VOUS)”*, when identified CNVs are novel, and may be associated with clinical phenotypes. The result of a VOUS offers a challenge to interpret and communicate to families [18]. Thus, the utility of CMA can only be ascertained by understanding the impact to families receiving these results, which is thus far unclear.

This gap in understanding the impact of CMA results to families highlights the limitation in the current conceptualization of *clinical utility*. Clinical utility of a biological test is defined on the basis of a set of criteria to be met prior to integrating that test into clinical practice [19] and has primarily been defined in the literature as benefits versus harms of a test on health outcomes in particular [20]. With the increased availability and access to genomics information, there is a need to expand the concept of “clinical utility” to overall utility that includes the utility of genomic information from the perspective of those affected by testing, regardless of its clinical use or health impact [20].

One way genomic information would have utility to individuals affected by testing is by increasing their knowledge of the condition, i.e. the extent to which a person can correctly identify facts from misconceptions about a condition. A systematic review found that genetic risk assessment services in cancer increases knowledge of the condition and of genetics [21]. In ASD, recent qualitative studies found that one of the outcomes of CMA in ASD is providing an etiological explanation to the condition to the parents [22–25]. This outcome could partly explain feeling *empowered* from attending clinical genetic services i.e. by increasing a sense of control from having information [26]. In sum, knowledge of a condition may have an important role towards the overall utility of genomic testing specifically and biological testing in general. However, a major gap towards understanding the role of knowledge in overall utility is the absence of studies that have assessed knowledge of autism along with its predictors within a representative sample of families within the care pathway.

In this study, we examined knowledge of ASD among a representative sample of families undergoing clinically recommended CMA by assessing the potential predictors of knowledge, including sociodemographic factors, parental stress and distress, and time since diagnosis. Previous research has shown that higher levels of stress reduced the effectiveness of genetic risk counseling on improving risk comprehension among individuals at risk for a condition [27] while greater time since diagnosis of a condition consistently predicted more knowledge of the condition [28]. We thus hypothesized that lower parental stress and distress, and longer time since diagnosis would correlate with greater knowledge of ASD.

## Materials and methods

### Ethics statement

The study was approved by the Research Ethics Board of the McGill University Health Centre and the Research Ethics Board of the Douglas Mental Health University Institute. Written informed consent was obtained from participants. The study was performed in accordance with the Declaration of Helsinki.

### Participants

Recruitment of participants relied on a multi-site clinically integrated protocol as part of a larger longitudinal genomics study, *ASD Genome to Outcome* [with major genomic findings published in [29–32]]. Clinicians involved in the family’s clinical care introduced families who met the inclusion criteria to the research project. Inclusion criteria of the *ASD Genome to Outcome* study were: children or youth (aged 0–18 years) who were referred for an evaluation of ASD or a related neurodevelopmental condition, or had a confirmed diagnosis of ASD or a related condition for which CMA was recommended. Exclusion criteria for the *ASD Genome to Outcome* study were: children with previously diagnosed genetic disorders (e.g. chromosomal or cytogenetic abnormalities, such as Trisomy 21, Duchenne Muscular Dystrophy, Angelman Syndrome, William’s Syndrome, etc.). The current study included a sub-sample of the ASD Genome to Outcome study, specifically only the individuals who were diagnosed with ASD. There were no other exclusion criteria.

Enrolled families provided informed written consent. The caregiver “most knowledgeable” about the child was asked to complete online questionnaires at home. A subset of the questionnaires was completed during the study visit to help respondents familiarize themselves to the format of the online questionnaires.

**Fig 1:**
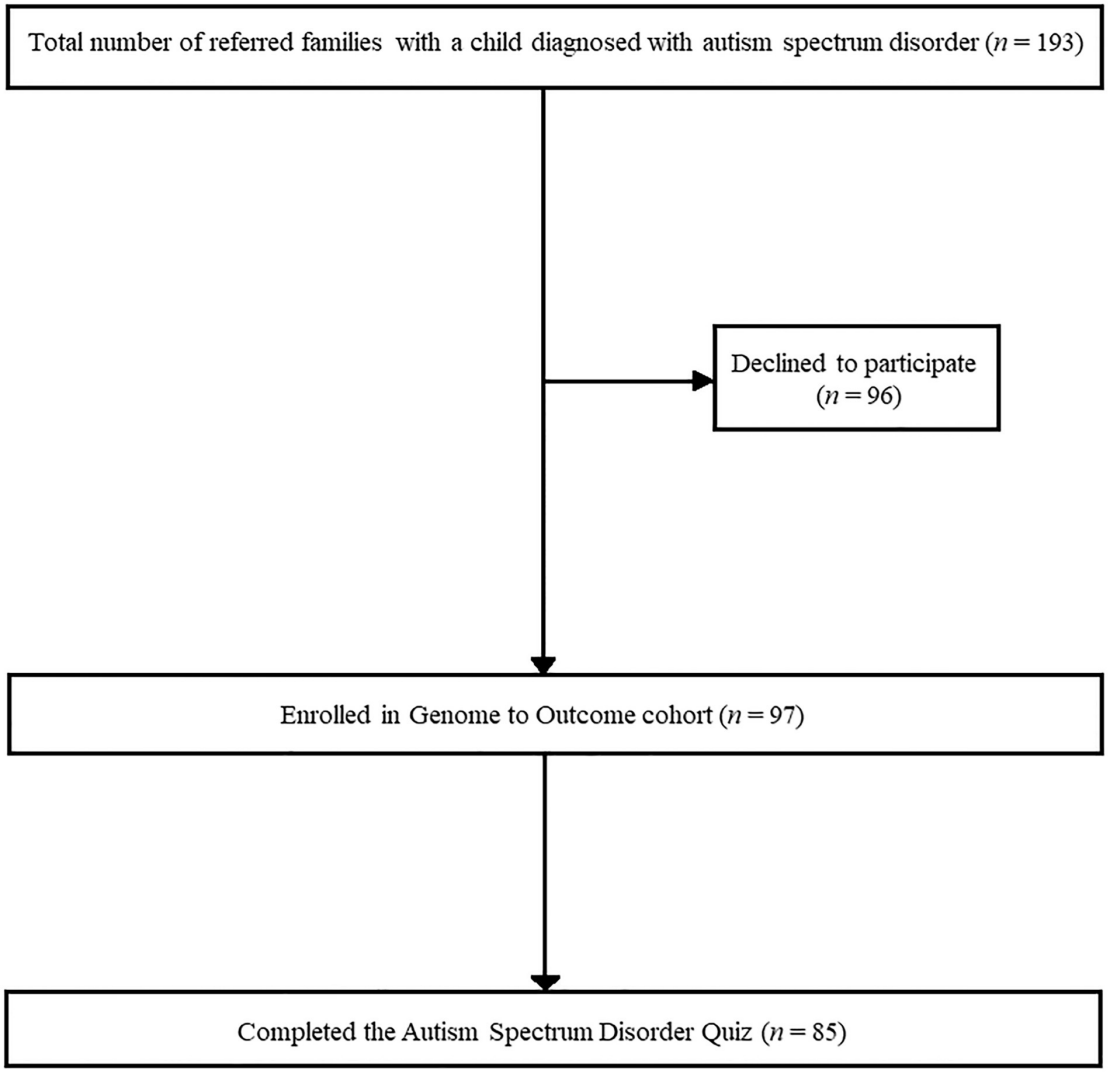
Flowchart of enrollment into the study.

Fig 1 outlines the inclusion of participants into the current study. A total of 193 eligible families with a child diagnosed with ASD were assessed for interest in research participation by their clinician between 2016 and 2018 (Fig 1). Paediatricians referred the most families (*n* = 92/193, 48%). Ninety seven families agreed to participate (50%).

The demographics of enrolled families are detailed in Table 1. The majority of respondents were married or common-law and were biological mothers to a male child. Forty-two percent of the respondents had a high school or college diploma as their highest completed degree and approximately one-third of the families reported an annual household income of less than $40,000. The median time since diagnosis for the enrolled families was 150 days, with a range of 16 days to 13 years and 4 months.

**Table 1:** Characteristics of enrolled families (*n* = 97).

Characteristic	Statistic
Respondent age in years *M* (*SD*)	39.0 (7.7)
Respondent’s relationship to child *N* (%)	
Biological mother	90 (92.8)
Biological father	7 (7.2)
Marital status *N* (%)	
Married/common law	81 (83.5)
Single/separated/divorced	16 (16.5)
Respondent education background *N* (%)	
Diploma or certificate below bachelor level°	42 (43.3)
Bachelor’s degree or higher	55 (57.3)
Annual household income *N* (%)	
Less than $40,000	28 (28.9)
Between $40,000 and $80,000	30 (30.9)
More than $80,000	38 (39.2)
Missing	1(1.0)

Note.

°This includes a diploma/certificate from High School, Community College, CEGEP or Nursing School or University, or Trade, Technical or Vocational School; SD: Standard deviation

### The Autism Spectrum Disorder (ASD) Quiz

Assessing knowledge in the context of biological testing requires assessing knowledge of the neurobiology of ASD and the feasibility of biological testing in ASD. In the ASD field, few knowledge instruments are validated, and existing validated knowledge instruments primarily concern with knowledge of ASD features and development [33]. Thus, we developed this ASD Quiz to incorporate concepts related to neurobiology of ASD and the feasibility of biological testing in ASD. Briefly, guided by established methodology [34, 35], the development of the ASD Quiz consisted of first, an initial generation of 12 items on the heritability of ASD and the feasibility of biological testing in ASD integrated with items retrieved and adapted from a literature review of questionnaires assessing parents’ knowledge of genetics and heritability of any neurodevelopmental condition. Following expert validation of items for accuracy and clarity and translation of the questionnaire to French, we conducted cognitive interviews among parents from the target population. Each step in the development process is detailed in S1 Text.

The questionnaire items are provided in S1 Table. The ASD Quiz is composed of 19 statements rated either *True* or *False*. A knowledge score was calculated as the percent of correct statements chosen. Higher scores reflect greater knowledge of ASD.

#### Validity and reliability of the ASD Quiz

Cognitive interviews suggested overall good face validity of the ASD Quiz for the target population: the questionnaire has adequate readability, with parents reporting consistent understanding of the items of the questionnaire.

**Fig 2:**
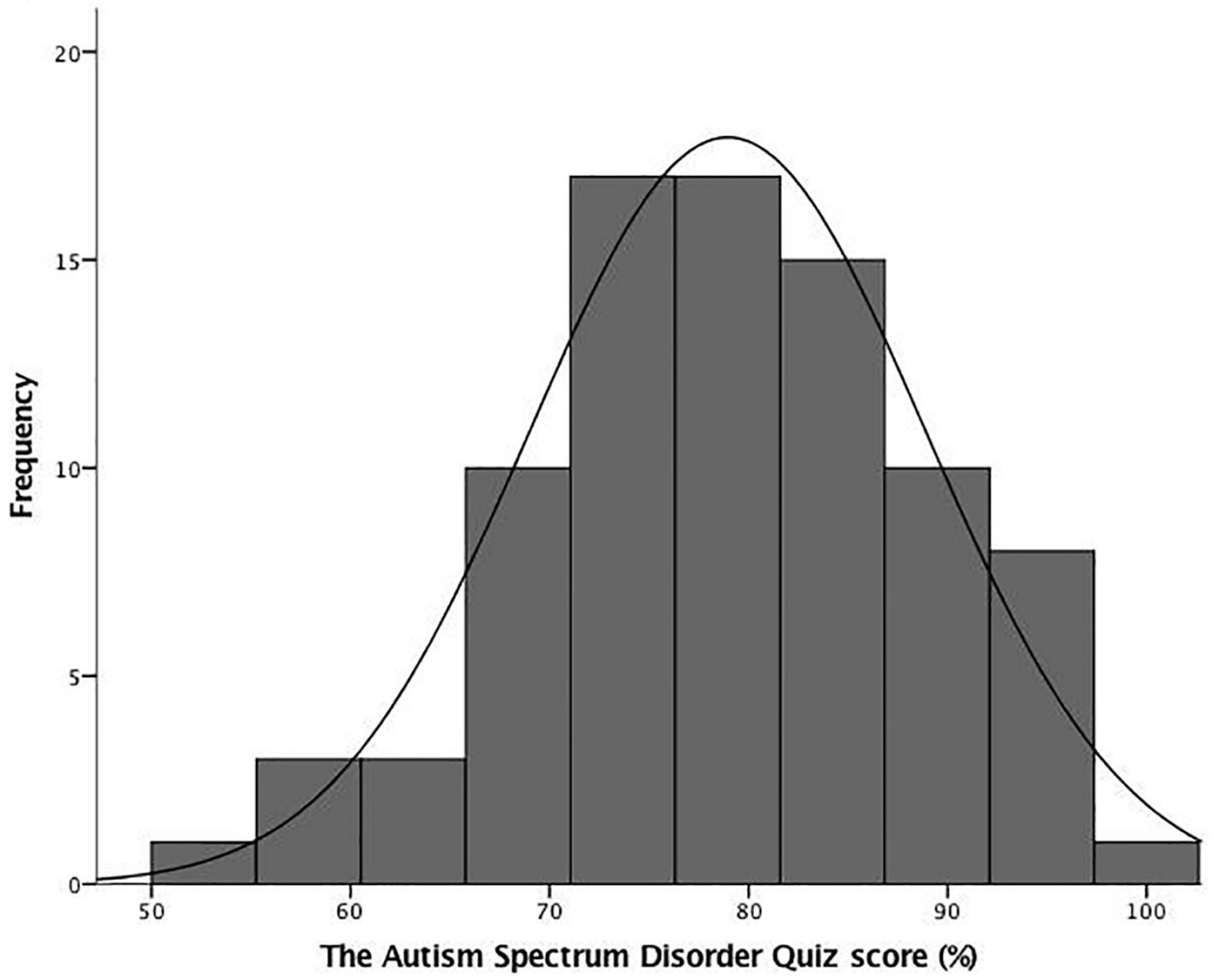
Histogram of the Autism Spectrum Disorder scores.

A total of 85 out of 97 enrolled families completed the quiz, a response rate of 88%. This indicates a high rate of return and thus minimizes the possibility of non-response bias. The item-by-item responses in the quiz are summarized in the S1 Table. Fig 2 is a histogram of quiz scores, which demonstrates a negative skewed distribution. This suggests a possible ceiling effect of knowledge among parents of a child on the autism spectrum.

The long-interval temporal stability of the ASD Quiz is supported by the significant correlation between ASD Quiz scores at baseline versus after chromosomal microarray results became available, *Pearson’s r* = 0.58, *p* < 0.001 (*n* = 41). The average time between the two time-points was 25.1 weeks (*SD* = 10.3, *Min* = 3.3, *Max* = 51.0).

### Correlates of knowledge of ASD

The correlates of knowledge assessed are as follows: parental stress and distress, time since diagnosis, and sociodemographic factors, such as parental age and education, child age and gender, and annual household income. Measures pertaining to each of the correlates are detailed next.

Parent stress was measured using the 10-item version of the Perceived Stress Scale (PSS-10) [36]. It measures the extent to which situations in one’s life in the past month are perceived as stressful. The PSS-10 has been previously shown to have high internal reliability in a sample of the general population (coefficient alpha = 0.85), adequate test-retest reliability, is correlated with life-event scores, and is a better predictor of health and health-related outcomes than life-event scores [36]. Higher scores indicate higher levels of parental stress.

Parent distress was measured using the distress thermometer (DTh) [37]. The DTh was developed “to detect distress in parents of a chronically ill child”. The DTh has been shown to have good internal consistency (Cronbach alpha ≥ 0.90). The DTh was able to predict clinical levels of anxiety and depression and was associated with parental stress. [37]. Higher scores on the DTh reflect higher parental distress.

Sociodemographic variables of interest are: respondent age, education, and annual household income. These variables were assessed using the Family Background Information Questionnaire (FBIQ) [38].

## Results

### Independent relationships with knowledge

**Fig 3:**
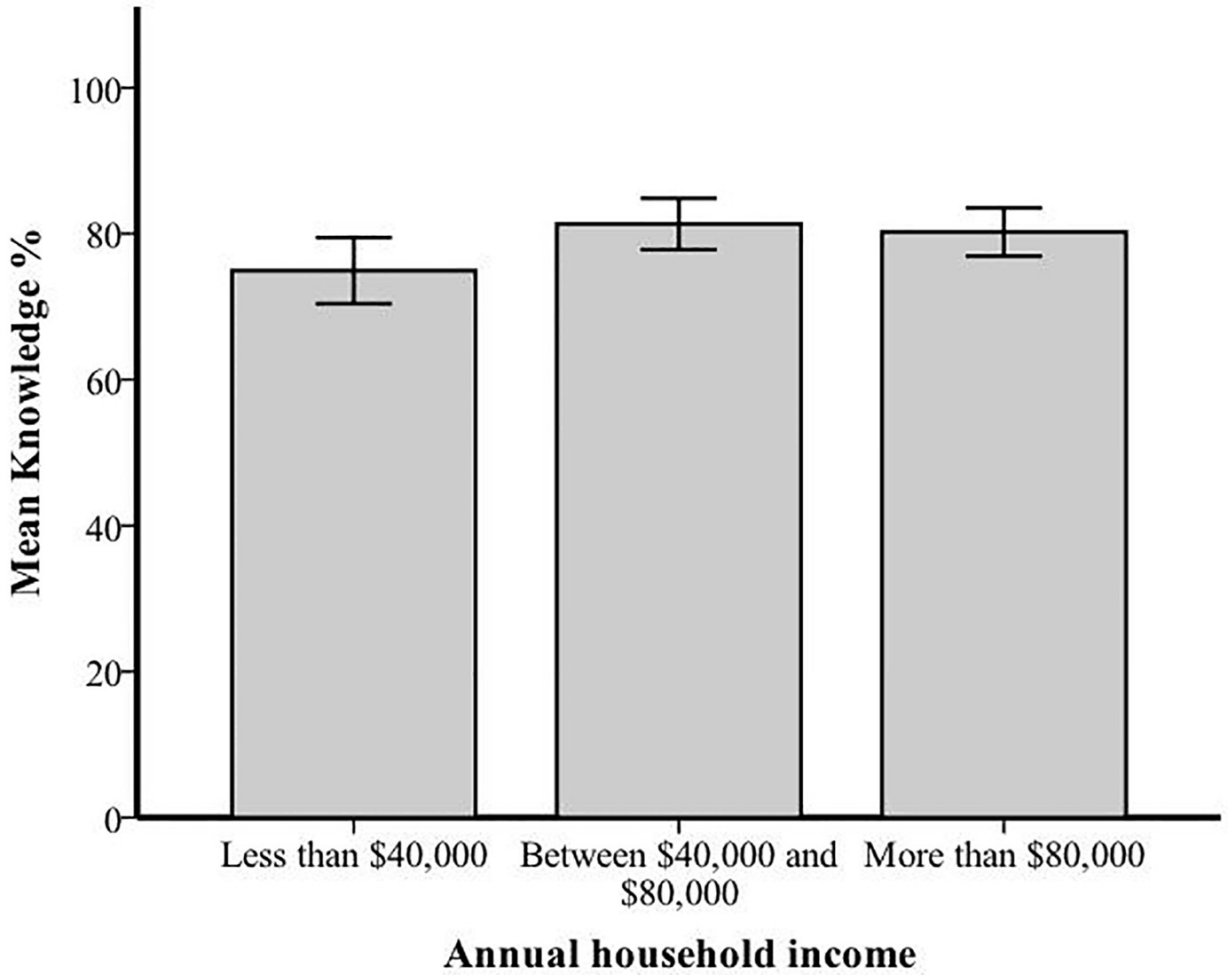
Mean knowledge score (%) for each annual household income level. Error bars represent standard errors.

We first assessed the extent to which each of the sociodemographic variables is independently associated with knowledge of ASD. The extent to which knowledge differs by child and parent age was assessed using Pearson’s r correlation, and the relationship between time since diagnosis and knowledge was assessed with a Spearman’s ρ correlation. The effect of parent education level, household income, and whether or not families had already undergone genetic testing for ASD on knowledge was examined using independent-samples *t* tests or a one-way analysis of variance, when applicable. None of the factors significantly correlated with knowledge (Table 2). The average knowledge score was 6.4% and 5.3% lower in families reporting an annual household income of less than $40,000 compared to those reporting between $40,000 and $80,000 and those reporting more than $80,000, respectively. While, these differences showed a trend towards statistical significance (*p* = 0.052), they were not significant based on the *p* = 0.05 threshold (Table 2, Fig 3).

**Table 2:** Sociodemographic variables and time since diagnosis as independent correlates of knowledge.

Variable	*n*	Mean Knowledge % (SD)	Statistic	*p*-value
Child age	85	-	Pearson’s *r* = -0.01	0.92
Child gender	85	-	*t* (83) = -0.41	0.68
Female	19	78.1 (11.4)	-	-
Male	66	79.2 (9.6)	-	-
Parent age	85	-	Pearson’s *r* = -0.07	0.52
Parent education level	85	-	*t* (83) = 1.52	0.13
Diploma or certificate below bachelor level	36	77.0 (9.8)	-	-
Bachelor’s degree or higher	49	80.3 (9.9)	-	-
Household income	84	-	*F* (2, 81) = 3.08	0.052
Less than $40,000	25	74.9 (11.0)	-	-
Between $40,000 and $80,000	22	81.3 (7.9)	-	-
More than $80,000	37	80.2 (9.9)	-	-
Have undergone genetic testing for ASD	85	-	*t* (83) = 0.13	0.90
Yes	25	79.2 (10.6)	-	-
No	60	78.9 (9.8)	-	-
Time since diagnosis	63	-	Spearman’s *ρ* = -0.001	1.00

*Note*. ASD: Autism spectrum disorder; SD: Standard deviation

The descriptive statistics of potential predictors are presented in Table 3. The relationship between parent stress, parent distress, and knowledge was each assessed using Pearson’s *r* (Table 3). Higher scores on the DTh and on the PSS-10 were moderately associated with higher scores on the ASD Quiz. PSS-10 was strongly correlated with DTh.

**Table 3:** Descriptive statistics of potential correlates of autism knowledge along with bivariate correlations between factors and knowledge.

Variable	N	Min-Max	M (SD)	Pearson’s *r* with Knowledge score	Pearson’s *r* with PSS-10
PSS-10 total score	84	5–32	17.9 (7.0)	0.26*	-
DTh	84	0–9	4.2 (2.8)	0.30*	0.70*

*Note*. PSS-10: Perceived Stress Scale 10-item version; DTh: Distress Thermometer

*p < 0.05

### Multiple regression model

To explore the combined effect of correlates on knowledge, we entered the following as predictors of interest in a stepwise regression model predicting knowledge: respondent age, education, household income, whether or not the family has undergone genetic testing for ASD, time since diagnosis, parental stress, and parental distress.

The best-fitting model accounted for 7.8% of the variance in knowledge, *F* (1, 82) = 8.02, *p* = 0.006, *R*^*2*^*-adjusted* = 0.078. The predictor significant to knowledge was parental distress, β = 0.30, *t* = 2.83, *p* = 0.006. Specifically, higher distress is moderately associated greater knowledge. No other factors were associated with ASD knowledge.

## Discussion

We examined parents’ knowledge of ASD among a relatively large number of families mostly recruited from routine diagnostic or medical care services. Our results showed that, in a constraint model, higher parental distress correlated with greater ASD knowledge. We did not find an association between knowledge with parental stress or time since diagnosis. Further, socio-demographic factors, namely parental education, and parent age and gender, were not associated with knowledge. The association between income and knowledge showed a trend towards significance.

In contrast to the hypothesis, distress positively correlated with baseline knowledge of biological testing in ASD. Past studies have shown that greater distress predicts more help-seeking [39]. It is possible that parents with greater distress have also sought more information on ASD because of this help-seeking behavior. Alternatively, it is also possible that greater knowledge leads to greater distress because more unmet concerns are generated. This would suggest that knowledge could have a detrimental impact if families lack appropriate support in navigating the needs generated by that knowledge. As such, empowering families with knowledge of both the condition and of the supports available are potentially key to reducing distress by improving acceptance of the condition: mothers who recalled having a higher *confidence* in knowledge of ASD in general at the time of their child’s ASD diagnosis also recalled positive feelings for their children [40]. Further research assessing both help-seeking behavior along with quality of care in examining the relationship between distress and knowledge is needed to inform this question.

Our results signify the need to control for both baseline knowledge and distress in studies examining the effect of an educational intervention in genetic testing. Specifically, it is possible that those present with higher distress also had higher baseline knowledge, which would be less likely to improve after an intervention due to a ceiling effect. This may explain previous results on the effect of worry on “reducing” the effectiveness of a genetics risk intervention [27].

### Limitations and future directions

To ensure face validity, the ASD Quiz was developed with input from experts and families in addition to being informed by the literature. We have also provided evidence of this measure’s stability. Future use of the ASD Quiz to distinguish the general population from ASD experts would provide further evidence of its validity. Other aspects of validity are also valuable, such as discriminant validity to examine if knowledge of ASD is distinct from knowledge of other concepts, like general genetics.

The questionnaires were implemented in a sample of families recruited via a clinically integrated protocol. The integrated protocol was successful in recruiting a more representative sample of families compared to previous research, as shown by the range of incomes and educational levels of families participating in the study. At the same time, there was a high rate of non-participation. We could not rule out the possibility that families who agreed to participate in a genetics project could have a higher knowledge than those who refused. Targeted recruitment and integrating the quiz in clinical assessments is important to address this possible bias.

The results of the study have implications in tailoring genetic counseling prior to undergoing testing. Parents who reported lower levels of distress could benefit from an information-driven counseling session, whereas those reporting higher levels of distress may profit from more psycho-emotional support during counseling rather than an information-focused session. Additionally, examining the role of income in moderating knowledge following genetic counseling should not be ruled out in the future, especially considering that socioeconomic status has been shown previously to affect parents’ access to services [41]. Further research among parents who have a child with a neurodevelopmental condition regarding how genetic counseling affects knowledge, distress, and perceived utility of biological testing, after controlling for potential moderators like socioeconomic status, similar to that done by Lerman et al. [27], is a needed next step in understanding how to improve the overall utility of biological testing in ASD and related conditions.

## Conclusions

The current study characterized the knowledge of biotesting in ASD among parents in routine care pathways for their child on the autism spectrum. We demonstrated how parents’ knowledge increases with higher parental distress. We concluded that families would require tailored support prior to undergoing genetic testing to address either knowledge gaps or high distress. With the advent of next-generation sequencing in standard care, parents are poised to receive more uncertainties in their genomics results. Ongoing appraisal of the informed consent process and its effects on the overall utility of biological testing among diverse backgrounds of families is necessary to ensure that optimal care is offered to families.

## Supporting information

S1 Table Items of the Autism Spectrum Disorder Quiz listed by percentage of participants correctly answering each item. (DOCX)

S1 Text Development of the Autism Spectrum Disorder Quiz. (DOCX)
