# Supplementary material for: Association between distress and knowledge among parents of autistic children
Source: PLoS One. 2019 Sep 26;14(9):e0223119. doi: 10.1371/journal.pone.0223119 (PMC6763195; doi:10.1371/journal.pone.0223119)
Supplement: S1 Table — (DOCX) [file pone.0223119.s001.docx]

**S1 Table. Items of the Autism Spectrum Disorder Quiz listed by percentage of participants correctly answering each item.**

| **Item** | **% Correct** |
| --- | --- |
| If one child in the family has autism then all other children in that family will develop autism too. *[false]* | 97 |
| Instead of one unique condition, there are probably many 'autisms' with different biological causes and different rates of developmental progress. *[true]* | 94 |
| The MMR vaccine causes autism. *[false]* | 93 |
| A doctor can use a genetic test to diagnose a child with autism instead of examining the child’s behaviour. *[false]* | 93 |
| With the proper treatment most children with autism eventually outgrow autism. *[false]* | 92 |
| A test can be done during pregnancy to find out whether or not the foetus will develop autism in the future. *[false]* | 92 |
| A genetic risk factor found in people with autism can never be found in people who developed typically. *[false]* | 89 |
| A genetic risk factor found in people with autism can also be found in people with other developmental disorders. *[true]* | 89 |
| Autism occurs in equal numbers among boys and girls. *[false]* | 88 |
| People with autism have a lesion in a specific part of the brain. *[false]* | 85 |
| Researchers know how genes and environment interact to cause autism. *[false]* | 84 |
| A genetic test can sometimes explain why a person has autism. *[true]* | 78 |
| Autism changes the way the brain develops. *[true]* | 74 |
| A doctor can use a genetic test to know which children in the community very likely have autism. *[false]* | 69 |
| A doctor can use a genetic test to identify possible medical issues in a child with autism. *[true]* | 69 |
| Scientists have found an “autism gene”. *[false]* | 62 |
| A doctor can use a genetic test to advise the family on the chances of their other children developing autism. *[true]* | 58 |
| A doctor can use brain imaging to find any neurological problems in children suspected of having autism. *[true]* | 48 |
| Many children who have genetic syndromes also have autism. *[true]* | 46 |
