# Supplementary material for: Association between distress and knowledge among parents of autistic children
Source: PLoS One. 2019 Sep 26;14(9):e0223119. doi: 10.1371/journal.pone.0223119 (PMC6763195; doi:10.1371/journal.pone.0223119)
Supplement: S1 Text — (DOCX) [file pone.0223119.s002.docx]

# Development of the Autism Spectrum Disorder Quiz

The development of the Autism Spectrum Disorder (ASD) Quiz followed established methodology [1, 2], consisting of item generation integrating expert input and literature review, linguistic adaptation, and cognitive interviewing. Each step in the development process is detailed next.

**Item generation**

Two authors (AY and ME) generated items guided by two main concepts: the heritability of ASD and the feasibility of biological testing in identifying ASD in different contexts. We generated 12 items in this initial stage.

We then reviewed the literature on parents’ knowledge of genetics and heritability of neurodevelopmental conditions. We retrieved questionnaire items from 22 articles. These articles assessed knowledge related to the following conditions: Autism (*n* = 7) [3-9], Batten disease (*n* = 1) [10], breast cancer (*n* = 1) [11], Cystic Fibrosis (*n* = 4) [12-15], Down's syndrome (*n* = 1) [16], Inherited Bone Marrow Failure Syndromes (*n* = 1) [17], Sickle Cell (*n* = 2) [18, 19], Neurofibromatosis Type 1 (*n* = 1) [20], Attention Deficit Hyperactivity Disorder (*n* = 1) [21], Hemochromatosis (*n* = 1) [22], general psychiatry (*n* = 1) [23], and Parkinson's Disease (*n* = 1) [24].

When available, psychometric properties of the tools were also reviewed. All items identified from the questionnaires were compiled into an item bank. AY and ME reviewed each item for relevance for biological testing in ASD. Ten relevant items from the review plus the 12 items generated by the authors were compiled into a single questionnaire tool and adapted for the target population of parents of children undergoing genetic testing, e.g., modifications of relevant questions relating to the application of biological testing in ASD and changing phrasing of some questions. Expert validation was conducted as part of the adaptation: a medical geneticist and a genetics counsellor reviewed the items for accuracy and clarity.

**Linguistic adaptation**

Considering that the questionnaire was intended for use with a sample of families in Quebec, a French-language version was also developed based on established adaptation guidelines [25]. A French-English bilingual clinical researcher translated the questionnaires into Quebec French. They were then back-translated into English by another bilingual clinical researcher, blind to the original questionnaires. The two translators compared the English versions to reconcile discrepancies in the questionnaires. Items were revised or regenerated as needed. The translated questionnaires were finally revised by a bilingual health professional for the final version of the French version.

**Follow up cognitive interviews**

To examine the face validity of the questionnaire, we conducted a series of follow-up cognitive interviews [26] with a sub-sample of parents from the target population to verify that the terms were interpreted consistently across participants. Research on cognitive interviewing as an approach to establishing questionnaire validity has argued that cognitive interviewing is crucial in ensuring relevance and clarity of questionnaire items that would be problematic otherwise [27]. In this study, respondents were asked to verbalize their thought process while reading the questionnaire items, with the help of verbal probing. Cognitive interviews were completed in-person, after the respondent had completed the online questionnaire at home. The interview was audio-recorded and transcribed off-line. During the interview, probes were used to ensure that all participants were asked the same question consistently. Probes for each item included: (1) *Can you repeat the statement in your own words?* (2) *How did you arrive at that answer?* and (3) *Was this hard or easy to answer?*

Seven mothers from the overall sample participated in the cognitive interview. All mothers have a boy with ASD. The median age of the mothers is 35.1 years (range=28-45). The median age of their child with ASD is 5.8 years old (range=5-11). Most mothers had a Bachelor’s degree or higher, and all mothers reported an annual household income of more than $80,000.

## Summary of development and validation of the ASD Quiz

In summary, the development of the ASD Quiz followed established guidelines. Items were generated based on the literature and integrating expert review, ensuring content validity. Cognitive interviews confirmed the face validity. There was adequate long-term temporal stability. Thus, we contend that the total ASD Quiz score reflects knowledge of ASD.

**References**

1. US Department of Health and Human Services Food and Drug Administration. Guidance for industry: patient-reported outcome measures: use in medical product development to support labeling claims. . US Department of Health and Human Services Food and Drug Administration; 2009.

2. Aaronson N, Alonso J, Burnam A, Lohr KN, Patrick DL, Perrin E, et al. Assessing health status and quality-of-life instruments: attributes and review criteria. Quality of life research : an international journal of quality of life aspects of treatment, care and rehabilitation. 2002;11(3):193-205.

3. Farmer J, Reupert A. Understanding Autism and understanding my child with Autism: An evaluation of a group parent education program in rural Australia. Australian Journal of Rural Health. 2013;21(1):20-7 8p.

4. Kuhn JC, Carter AS. Maternal self-efficacy and associated parenting cognitions among mothers of children with autism. The American journal of orthopsychiatry. 2006;76(4):564-75.

5. Johnson N, Van Hecke A. Increasing Autism Awareness in Inner-City Churches: A Brief Report. Journal of pediatric nursing. 2015;30(6):e63-9.

6. Zuckerman KE, Sinche B, Cobian M, Cervantes M, Mejia A, Becker T, et al. Conceptualization of autism in the Latino community and its relationship with early diagnosis. Journal of Developmental & Behavioral Pediatrics. 2014;35(8):522-32.

7. Harrington JW, Rosen L, Garnecho A, Patrick PA. Parental perceptions and use of complementary and alternative medicine practices for children with autistic spectrum disorders in private practice. Journal of developmental and behavioral pediatrics : JDBP. 2006;27(2 Suppl):S156-61.

8. Mickelson KD, Wroble M, Helgeson VS. “Why My Child?”: Parental Attributions for Children's Special Needs1. Journal of Applied Social Psychology. 1999;29(6):1263-91.

9. Stone WL. Cross-Disciplinary Perspectives on Autism1. Journal of Pediatric Psychology. 1987;12(4):615-30.

10. Adams HR, Rose K, Augustine EF, Kwon JM, deBlieck EA, Marshall FJ, et al. Experience, knowledge, and opinions about childhood genetic testing in Batten disease. European journal of clinical nutrition. 2014;111(2):197-202.

11. Erblich J, Brown K, Kim Y, Valdimarsdottir HB, Livingston BE, Bovbjerg DH. Development and validation of a breast cancer genetic counseling knowledge questionnaire. Patient education and counseling. 2005;56(2):182-91.

12. Baker HM, Brown RL, Tluczek A. Development and validation of a cystic fibrosis genetic knowledge questionnaire within the general population of the United States. Journal of cystic fibrosis : official journal of the European Cystic Fibrosis Society. 2013;12(5):504-11.

13. Ciske DJ, Haavisto A, Laxova A, Rock LZ, Farrell PM. Genetic counseling and neonatal screening for cystic fibrosis: an assessment of the communication process. Pediatrics. 2001;107(4):699-705.

14. Braekeleer MD, Bellis G, Rault G, Allard C, Milot M, Simard F. Disease knowledge in a high-risk population for cystic fibrosis. Patient education and counseling. 2001;43(3):263-8.

15. McClaren BJ, Aitken M, Massie J, Amor D, Ukoumunne OC, Metcalfe SA. Cascade carrier testing after a child is diagnosed with cystic fibrosis through newborn screening: investigating why most relatives do not have testing. Genet Med. 2013;15(7):533-40.

16. Seidenfeld MJ, Braitman A, Antley RM. The determinants of mothers' knowledge of the Down syndrome before genetic counseling: part II. Am J Med Genet. 1980;6(1):9-23.

17. Hamilton JG, Hutson SP, Frohnmayer AE, Han PKJ, Peters JA, Carr AG, et al. Genetic Information-Seeking Behaviors and Knowledge among Family Members and Patients with Inherited Bone Marrow Failure Syndromes. Journal of genetic counseling. 2015;24(5):760-70.

18. Acharya K, Lang CW, Ross LF. A pilot study to explore knowledge, attitudes, and beliefs about sickle cell trait and disease. Journal of the National Medical Association. 2009;101(11):1163-72.

19. Dormandy E, Tsui EY, Marteau TM. Development of a measure of informed choice suitable for use in low literacy populations. Patient Education & Counseling. 2007;66(3):278-95 18p.

20. Benjamin CM, Colley A, Donnai D, Kingston H, Harris R, Kerzin-Storrar L. Neurofibromatosis type 1 (NF1): knowledge, experience, and reproductive decisions of affected patients and families. Journal of medical genetics. 1993;30(7):567-74.

21. Bussing R, Gary FA, Mills TL, Garvan CW. Cultural variations in parental health beliefs, knowledge, and information sources related to attention-deficit/hyperactivity disorder. Journal of Family Issues. 2007;28(3):291-318 28p.

22. McLaren CE, Barton JC, Adams PC, Harris EL, Acton RT, Press N, et al. Hemochromatosis and Iron Overload Screening (HEIRS) Study Design for an Evaluation of 100,000 Primary Care-Based Adults. The American Journal of the Medical Sciences. 2003;325(2):53-62.

23. Laegsgaard MM, Kristensen AS, Mors O. Potential consumers' attitudes toward psychiatric genetic research and testing and factors influencing their intentions to test. Genetic testing and molecular biomarkers. 2009;13(1):57-65.

24. Falcone DC, Wood EM, Xie SX, Siderowf A, Van Deerlin VM. Genetic Testing and Parkinson Disease: Assessment of Patient Knowledge, Attitudes, and Interest. Journal of genetic counseling. 2011;20(4):384-95.

25. Wild D, Grove A, Martin M, Eremenco S, McElroy S, Verjee-Lorenz A, et al. Principles of Good Practice for the Translation and Cultural Adaptation Process for Patient-Reported Outcomes (PRO) Measures: Report of the ISPOR Task Force for Translation and Cultural Adaptation. Value in Health. 2005;8(2):94-104.

26. Tourangeau R. Cognitive sciences and survey methods. Cognitive aspects of survey methodology: Building a bridge between disciplines. 1984:73-100.

27. Collins D. Pretesting survey instruments: An overview of cognitive methods. Quality of Life Research. 2003;12(3):229-38.
